# Predictors of hospital and one-year mortality in intensive care patients with refractory status epilepticus: a population-based study

**DOI:** 10.1186/s13054-017-1661-x

**Published:** 2017-03-23

**Authors:** Anne-Mari Kantanen, Reetta Kälviäinen, Ilkka Parviainen, Marika Ala-Peijari, Tom Bäcklund, Juha Koskenkari, Ruut Laitio, Matti Reinikainen

**Affiliations:** 1 Epilepsy Center, Neurocenter, Department of Neurology, Kuopio University Hospital, Kuopio, Finland; 2 Epilepsy Center, Neurocenter, Department of Neurology, Kuopio University Hospital and Faculty of Health Sciences, School of Medicine, Institute of Clinical Medicine, University of Eastern Finland, Box 100, Kuopio, 70029 KYS Finland; 3 Department of Intensive Care, Kuopio University Hospital and Faculty of Health Sciences, School of Medicine, Institute of Clinical Medicine, University of Eastern Finland, Kuopio, Finland; 4 Division of Intensive Care, Tampere University Hospital, Tampere, Finland; 5 Departments of Internal Medicine, Helsinki University Hospital, Helsinki, Finland; 6 Department of Anesthesiology, Division of Intensive Care Medicine, Oulu University Hospital, Oulu, Finland; 7 Division of Perioperative Services, Intensive Care and Pain Medicine, Turku University Hospital, Turku, Finland; 8 Department of Intensive Care, North Karelia Central Hospital and Faculty of Health Sciences, School of Medicine, Institute of Clinical Medicine, University of Eastern Finland, Joensuu, Finland

**Keywords:** Status epilepticus, Refractory status epilepticus, Super-refractory status epilepticus, Incidence, Mortality, ICU treatment, Outcome

## Abstract

**Background:**

The aim was to determine predictors of hospital and 1-year mortality in patients with intensive care unit (ICU)-treated refractory status epilepticus (RSE) in a population-based study.

**Methods:**

This was a retrospective study of the Finnish Intensive Care Consortium (FICC) database of adult patients (16 years of age or older) with ICU-treated RSE in Finland during a 3-year period (2010–2012). The database consists of admissions to all 20 Finnish hospitals treating RSE in the ICU. All five university hospitals and 11 out of 15 central hospitals participated in the present study. The total adult referral population in the study hospitals was 3.92 million, representing 91% of the adult population of Finland. Patients whose condition had a post-anoxic aetiological basis were excluded.

**Results:**

We identified 395 patients with ICU-treated RSE, corresponding to an annual incidence of 3.4/100,000 (95% confidence interval (CI) 3.04–3.71). Hospital mortality was 7.4% (95% CI 0–16.9%), and 1-year mortality was 25.4% (95% CI 21.2–29.8%). Mortality at hospital discharge was associated with severity of organ dysfunction. Mortality at 1 year was associated with older age (adjusted odds ratio (aOR) 1.033, 95% CI 1.104–1.051, *p* = 0.001), sequential organ failure assessment (SOFA) score (aOR 1.156, CI 1.051–1.271, *p* = 0.003), super-refractory status epilepticus (SRSE) (aOR 2.215, 95% CI 1.20–3.84, *p* = 0.010) and dependence in activities of daily living (ADL) (aOR 2.553, 95% CI 1.537–4.243, *p* < 0.0001).

**Conclusions:**

Despite low hospital mortality, 25% of ICU-treated RSE patients die within a year. Super-refractoriness, dependence in ADL functions, severity of organ dysfunction at ICU admission and older age predict long-term mortality.

**Trial registration:**

Retrospective registry study; no interventions on human participants.

## Background

Status epilepticus (SE) is a neurological emergency that may cause death and marked neurological deficiency. SE is called refractory status epilepticus (RSE) if the first-line and second-line medication does not terminate the seizure, and it is referred to super-refractory SE (SRSE) if it continues over 24 h after the onset of the first anaesthesia [1]. Less than 50% of patients with SE have had previous seizures or epilepsy. There are several other aetiologies, but the cause is usually an underlying acute neurological disease, systemic disorder or condition with a remote aetiological basis after previous central nervous system injury [2, 3].

Data on the long-term outcomes of SE are scarce, particularly in the cases of RSE and SRSE, and they are based on small patient cohorts [4]. Age, aetiology of disease, type of seizure at onset, treatment delay, level of consciousness at presentation, use of electroencephalography (EEG) and delay in admission to the intensive care unit (ICU) are reported to affect outcomes [4–7]. In-hospital seizures seem to have worse outcomes than those starting outside the hospital setting [7, 8]. Moreover, there are both national and international guidelines and algorithms for better treatment of SE [2, 9–13]; however, the outcomes remain poor: Short-term mortality in adult SE and RSE vary in the range of 19–39% [6, 14], while longer-term mortality is in the range of 35–43% [4, 15]. Long-term mortality in SRSE is twofold higher than in RSE alone [16]. The mortality of SE is correlated with seizure duration, rapid identification of SE and aetiology of disease [1].

Our study group has recently reported on the incidence of SRSE and the mortality rates in patients with SRSE from this same patient cohort [16]. The aim of the present study was to determine predictors of hospital and 1-year mortality in ICU-treated patients with RSE, including patients evolving to SRSE, in a nationwide population-based study.

## Methods

### Data source

The Finnish Intensive Care Consortium (FICC) is a body responsible for a national ICU benchmarking programme and database in Finland. All 20 major central and university hospitals, providing all secondary and tertiary care, ICUs and neurological services for their referral population in Finland, joined the consortium by 2007. The FICC database collects data from every ICU admission from all general adult ICUs in all 20 Finnish hospital districts.

Information on clinical characteristics, severity of illness and outcomes is collected in the database; this is validated by each participating ICU before submission to the central database [17]. All 5 university hospitals and 11 out of 15 central hospitals participated in the present study. Both ICU physicians and neurologists participate in the diagnostics and patient care in these ICUs. We used the FICC database and medical records to identify adult patients (age 16 years or over) with RSE treated with general anaesthesia in the ICU) in a population-based cohort in Finland during the 3-year period of 2010–2012. This study received FICC Board approval to access the database, while the authorities responsible for specific hospital districts authorised the use of medical records data.

### Patients

**Fig. 1:**
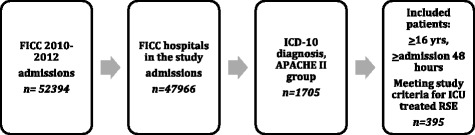
Data collection flowchart. The Finnish Intensive Care Consortium (*FICC*) database had 52,394 ICU admissions in 2010–2012, and 395 patients met the study criteria of intensive care unit (ICU) general anaesthesia-treated refractory status epilepticus (*RSE*). *APACHE* acute physiology and chronic health evaluation

We included consecutive adult patients (age 16 years or older) with RSE treated with general anaesthesia. To identify patients treated in an ICU for seizure disorders, we first searched the FICC database for patients who had been classified under the acute physiology and chronic health evaluation (APACHE) II diagnostic group “seizure”’ [18] or for whom one of the ICD-10 codes for epilepsy, SE or convulsions (G40.X, G41.X or R56.8) had been documented. We only included patients who had been treated in the ICU for at least 48 h, which we estimated to be the minimum duration of treatment and weaning period for patients with RSE treated with general anaesthesia. Intensive care unit physicians at each participating hospital re-evaluated the patients’ medical records to identify patients with RSE, that is, patients who had prolonged seizures that did not improve with first-line and second-line treatment with antiepileptic drugs (AEDs) and were treated with general anaesthesia in the ICU. Patients with post anoxic aetiologies were excluded. Altogether 395 incidents of RSE fulfilled the final inclusion criteria (Fig. 1).

### Clinical factors

The simplified acute physiology score II (SAPS II) [19] and sequential organ failure assessment (SOFA) score [20] for the first 24 h in the ICU, describing the severity of organ failure and illness, were collected from the FICC database. Independence in activities of daily living (ADL) was coded as independent or non-independent (with supervision, direction or personal assistance) as a measure of each patient’s pre-morbid functional capacity. The Glasgow coma scale (GCS) for describing the impairment of consciousness was assessed at the time of ICU admission before any sedative medication was administered. Data on access to diagnostic EEG and continuous EEG monitoring during the ICU stay and data on the intravenous anaesthetics given were obtained from medical records by local intensive care physicians.

### Statistical analysis

Statistical analyses were conducted using SPSS software version 22 (IBM Corp, Armonk, NY, USA). We calculated the population incidence for RSE and 95% confidence intervals (CIs) for a single incidence rate. The chi-square test was used to compare the categorical variables, and nonparametric tests (Mann–Whitney *U* for test for the median) were used with continuous variables. Variables that were significant in univariate analyses were included in binary logistic multivariate regression analysis with the backward likelihood-ratio technique and significance variance exclusion set at 0.10.

## Results

### Patients and incidence

We identified 395 patients treated for RSE in the ICU during a 3-year period. The adult population of the participating 16 hospital districts is 3.92 million, representing 91% of the total Finnish adult population. The annual incidence of ICU-treated RSE was 3.4/100,000 (95% CI 3.04–3.71). Of the 395 patients, 264 (66.8%) were treated in university hospitals. The median age of the patients was 58 years (interquartile range (IQR) 46–67], and 245 (62.3%) of the patients were male. The median length of the ICU stay was 5.0 days (IQR 3.5–8), and the overall median hospital stay was 13 days (IQR 8–21). Altogether, 112 (28.2%) patients in the cohort needed help in their ADL functions before hospital treatment. Demographics and data are shown in Table 1.

**Table 1:** Demographics and clinical characteristics by hospital and one-year mortality

		By hospital outcome *n* = 394			By one-year outcome *n* = 390		
	All *n* = 395	Survivors *n* = 365	Non-survivors *n* = 29	*P* value	Survivors *n* = 291	Non-survivors *n* = 99	*P* value
Age, years, median (IQR)	58 (46–67)	58 (43–67)	62 (54–73)	0.033	55 (41.5–65)	62 (53–72.5)	<0.001
Male, *n* (%)	245 (62.3%)	229 (62.7%)	16 (55.2%)	0.41	185 (63.6%)	57 (57.6%)	0.25
Needing help in ADL, *n* (%)	112 (28.3%)	103 (28.2%)	9 (31.0%)	0.72	67 (23.0%)	44 (44.4%)	0.000037 < 0.001
SAPS II, median (IQR)	47 (36.5–57)	47 (36–57)	56 (47–60)	0.003	45 (35–55)	52 (43–61)	<0.001
SOFA, median (IQR)	8 (6–10)	8 (6–10)	10 (8–11)	0.005	8 (6–10)	9 (7–11)	0.003
SOFA score without CNS points, median (IQR)	5 (4–6)	5 (4–6)	6 (4.5–9)	0.005	5 (4–6)	5 (4–7)	0.013
GCS, median (IQR)	5 (4–10)	6 (4–10)	5 (3–7.5)	0.14	6 (4–10)	5 (3–8)	0.11
GCS <6, *n* (%)	195 (49.3%)	178 (48.8%)	17 (58.6%)	0.25	149 (51.2%)	42 (42.4%)	0.11
SRSE, *n* (%)	87 (22.0%)	78 (21.4%)	9 (31.0%)	0.15	54 (18.4%)	30 (30.3%)	0.011
Admission from ward, *n* (%)	71 (18.0%)	63 (17.3%)	8 (26.7%)	0.16	45 (15.5%)	25 (25.3%)	0.029
LOS ICU, median (IQR)	5.0 (3.5–8.0)	5.0 (3.5–7.8)	6.5 (4.1–10)	0.048	4.9 (3.2–7.8)	5.8 (3.9–9.1)	0.054
LOS hospital, median (IQR)	13 (8–21)	13 (8–22)	12 (8–19)	0.77	13 (8–21)	14 (9–22)	0.27
Hospital discharge							0.001
Home, *n* (%)	94 (23.8%)				88 (30.2%)	5 (5.0%)	
Specialist care hospital/ward, *n* (%)	86 (21.0%)				67 (23.0%)	15 (11.3%)	
Primary care hospital/ward, *n* (%)	185 (47.4%)				133 (45.7%)	52 (28.1%)	
Died in hospital, *n* (%)	29 (7.4%)						

*ADL* activities of daily living, *SAPS II* simplified acute physiology score II, *SOFA* sequential organ failure assessment, *GCS* Glasgow coma scale, *SRSE* super-refractory status epilepticus, *LOS* length of stay, *ICU* intensive care unit

### Diagnostics and treatment

Diagnostic EEG data were available for 186 patients (47.1%) and continuous EEG monitoring during anaesthesia was available for 313 (80.1%) of the RSE patients. Altogether, 39 patients (9.9%) had neither diagnostic nor continuous EEG monitoring. Propofol was given for 12–24 h as the first intravenous anaesthetic (IVA) in 373 of the patients (94.4%), whereas thiopental was administered for 12–24 h in 17 patients (4.3%). SRSE was identified in 87 patients (22.0%).

### Mortality

#### Hospital mortality

**Fig. 2:**
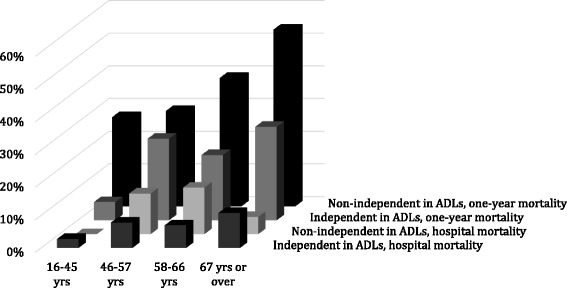
Hospital and one-year mortality by age quartiles and premorbid functional status. *ADLs* activities of daily living

The ICU mortality was 4/395 (1%, 95% CI 0–10.8%), and total hospital mortality was 29/395 (7.4%, 95% CI 0–16.9%). In multivariate regression analysis, the SOFA score was an independent predictor of hospital mortality. Neither age nor premorbid ADL stage predicted hospital mortality (Fig. 2, Table 2).

**Table 2:** Predictors of hospital and one-year mortality

Predictor	Univariate analysis			Multivariate analysis		
	OR	95% CI	*P* value	Adjusted OR	95% CI	*P* value
Hospital mortality						
Age	1.030	1.002–1.059	0.039			NS
Male gender	0.73	0.34–1.56	0.41			NS
Non-independence in ADL	1.16	0.51–2.66	0.72			NS
SOFA	1.25	1.09–1.44	0.002	1.22	1.06–1.41	*0.007*
GCS	0.93	0.83–1.04	0.18			NS
SRSE	1.83	0.79–4.24	0.16	2.04	0.86–4.84	0.104 NS
Admission from ward	1.83	0.77–4.31	0.17			NS
One-year mortality						
Age	1.04	1.02–1.06	<0.001	1.03	1.01–1.05	*<0.001*
Male gender	0.76	0.48–1.22	0.25			NS
Non-independence ADL	2.73	1.68–4.44	<0.001	2.55	1.46–4.13	*0.001*
SOFA	1.16	1.07–1.27	0.001	1.17	1.06–1.30	*0.002*
GCS	0.95	0.89–1.01	0.084			
SRSE	1.95	1.16–3.29	0.012	2.215	1.20–3.84	*0.010*
Admission from ward	1.84	1.06–3.20	0.031	1.73	0.94–3.2	0.08 NS

*OR* odds ratio, *CI* confidence interval, *ADL* activities of daily living, *SOFA* sequential organ failure assessment, *GCS* Glasgow coma scale, *SRSE* super-refractory status epilepticus, NS not significant. *P* values in italics are statistically significant

#### One-year mortality

A total of 366 (92.7%) patients were discharged from hospital. Only 23.8% of the patients were discharged to home. Most patients (47.4%) were discharged to primary healthcare wards and 21.0% to specialist care facilities. Ninety-nine patients (25.4%, 95% CI 21.2–29.8) died within 12 months of ICU admission. The discharge destination was highly predictive of outcomes at 12 months: only 5.4% of patients discharged to their home died, but mortality was 18.3% among those discharged to specialist care hospitals and 28.1% for patients discharged to primary care wards (*p* = 0.001). Mortality at 1 year was associated with older age, SRSE, severity of organ failure and lower premorbid capacity of ADL functions (Table 2). Both increasing age and poor premorbid functional performance were strong predictors of increased 1-year mortality (Fig. 2). Level of consciousness was measured by the GCS at presentation (median score 5 (IQR 4–10)), and there was no significant association with hospital mortality (*p* = 0.14) or 1-year mortality (*p* = 0.11).

## Discussion

Our study represents the first population-based, nationwide cohort showing the incidence and long-term mortality in ICU-treated and anaesthesia-treated RSE and SRSE. The incidence of RSE in our population-based cohort was 3.4/100,000/year, and the incidence SRSE in our cohort was 0.7/100,000/year [16], constituting 22% of the patients with RSE. Depending on the definition of RSE that was used, 4–26% of patients with RSE have previously been reported to develop SRSE [21]. According to Delaj et al. [21], if all patients with RSE are included, the frequency of SRSE is 4%, and if only intubated patients with RSE are included (as in our study), the frequency is 42%.

The rates of ICU mortality (1%) and hospital mortality (7%) in our study are comparable with recently reported rates of ICU mortality (2%) and hospital mortality (5%) in Australia and New Zealand [22], although this study included all prolonged seizures and not only RSE. The 1-year mortality rate (25%) in RSE in the present population-based study is relatively low compared to those reported in earlier studies, in which long-term mortality was in the rage of 26–62% (time variance of 1–12 months) [4, 23–26]. Consequently, the outcome in patients with RSE deteriorates after the period of stay in the ICU and the first weeks of hospitalisation, and one fourth of these patients die within a year.

We found that older age, SRSE, premorbid dependence in ADL and severity of organ dysfunction are associated with unfavourable long-term outcomes. Our data show that mortality doubles within 12 months if the patient needs help in ADL functions before the RSE incident and ICU admission. SOFA scores defining patient organ dysfunction and comorbidities are associated with poor long-term outcomes. Age and comorbidities have also been reported as measures of poor outcome in other studies of RSE [27–30]. The question remains as to whether it is the RSE, the aetiology of RSE, the patient’s pre-existing characteristics or the ICU treatment that explains the poor long-term outcome. This phenomenon is also seen in other ICU cohorts and studies of other diseases [31–33]. Age, comorbidities and frailty have major effects on the long-term outcomes of intensive care in general. In this study, level of consciousness measured by GCS at presentation to ICU did not predict mortality. This finding is in accordance with earlier studies by Sutter et al. [5, 34] in patients with RSE after excluding patients with hypoxic–ischaemic encephalopathy, as we have done in this cohort.

Consistency with the Finnish Current Care guideline was good in the choice of the first intravenous anaesthetic (IVA): propofol, the suggested drug, was used in 94% of patients [9]. Compliance with the guideline in access to diagnostic EEG and continuous EEG monitoring during anaesthesia was lower. Diagnostic EEG evaluation was used in less than half of the patients (47%), and continuous EEG monitoring was employed in 80% of patients. However, only 10% of the patients were treated without any EEG evaluation. RSE diagnostics should include EEG along with clinical judgement to differentiate between seizure activity, post seizure and medication-derived conditions and non-epileptic seizures (NES).

Patients can also be attended in the emergency department (ED) after having been sedated and intubated by paramedics and emergency doctors; therefore, evaluation of ongoing seizure activity and clinical presentation is necessary. Monitoring the depth of the coma and suppression of seizure activity, and secondarily, burst suppression (BS), is essential when titrating the depth of anaesthesia [9, 10].

Our study demonstrated that access to diagnostic EEG and continuous EEG monitoring during on-call hours is still not perfectly organised nationwide. This problem seems to be international; according to a recent article only 33% of the hospital trusts treating RSE in the UK had access to continuous EEG [35]. In our cohort, patients with no EEG data (10% of patients) were diagnosed as having RSE patients based on clinical judgement only. Unfortunately we have no data on AED management during the withdrawal of anaesthetics in the present study. This would be important because failure of the first anaesthesia and the occurence of SRSE may unnecessarily occur after inadequate use of background AEDs and not due to the refractoriness of the SE itself. Concomitant AED therapy should be started to prevent the recurrence of RSE, and it should be continued parallel to the acute emergency treatment to ensure adequate levels of AED medication both with intravenous preparations and via the nasogastric tube or percutaneus endoscopic gastrostomy if necessary. Patients with pre-existing epilepsy should have their previous antiepileptic medications continued with optimised doses [10].

ICU admission from a ward as compared to admission from the ED or other monitoring units was associated with 1-year mortality in univariate analysis. However, in our study there was no independent effect in multivariate analysis when adjusting for other risk factors. In some earlier studies hospitalised patients had RSE of more severe aetiology: focal brain abnormalities (stroke, tumour or trauma) or systemic metabolic disturbances had a worse outcome associated with the aetiology of the condition [8, 36]. In addition, as seizure duration over 1 h and especially over 24 h is associated with greater mortality in patients with RSE, a longer delay in treatment may be a factor in the poorer prognosis for patients with in-hospital RSE. Seizures are better recognised in the ED and monitoring units than in an in-patient ward setting [15, 37].

### Study limitations

A retrospective registry study has limitations: this nationwide study lacked information on the aetiology of RSE and long-term neurological outcomes apart from mortality. In addition, we did not have data on patients who may have had RSE but were treated outside of ICUs because of the presumed futility of intensive care. However, this is a large population-based study of 395 patients with ICU-treated RSE. The referral population of the participating hospitals represents over 90% of the Finnish population, and all major hospital districts and university hospitals with their catchment areas were included. We consider this study population highly representative of all patients with ICU-treated RSE in Finland.

## Conclusions

We conclude that ICU-treated RSE is a neurological emergency with a substantial 1-year mortality rate. Despite low mortality rates in the ICU and in hospital, 25% of the patients in our study died within a year. Older age, SRSE, premorbid non-independence in ADL functions and severity of organ failure predicted mortality at 1 year. Early effective treatment and preventing SE from developing into RSE would probably represent the best treatment actions to reduce mortality in RSE. Intensive care and general anaesthesia are the mainstays of treatment for RSE. Elderly or otherwise frail patients with impaired functional performance should be identified early and evaluated thoroughly individually; such patients should possibly be treated with a less aggressive method.

## Abbreviations

ADL: Activities of daily living
AED: Antiepileptic drug
APACHE: Acute physiology and chronic health evaluation
CI: Confidence interval
ED: Emergency department
EEG: Electroencephalography
FICC: Finnish Intensive Care Consortium
GCS: Glasgow coma scale
ICU: Intensive care unit
IQR: Interquartile range
IVA: Intravenous anaesthetic
RSE: Refractory status epilepticus
SAPS: Simplified acute physiology score
SE: Status epilepticus
SOFA: Sequential organ failure assessment
SRSE: Super-refractory status epilepticus
